# Frequency of Early Complications of Laparoscopic Sleeve Gastrectomy Using Four Ports

**DOI:** 10.7759/cureus.65613

**Published:** 2024-07-28

**Authors:** Rahman Ullah, Mashal Nazir, Nazia Shahana, Ibrahim Shuja, Muhammad A Fazal, Kainat Nazir, Fahad R Khan

**Affiliations:** 1 Surgical and Allied, Bacha Khan Medical Complex, Swabi, PAK; 2 Surgery, Police Services Hospital, Peshawar, PAK; 3 Surgery, Watford General Hospital, Watford, GBR; 4 Surgery, Khyber Teaching Hospital, Peshawar, PAK; 5 Cardiology, Lady Reading Hospital, Peshawar, PAK

**Keywords:** postoperative outcomes, bariatric surgery, four-port technique, early complications, laparoscopic sleeve gastrectomy (lsg)

## Abstract

Background

Laparoscopic sleeve gastrectomy (LSG) has become a widely accepted bariatric procedure for treating morbid obesity and associated comorbidities due to its relatively straightforward technique and positive outcomes in terms of weight loss and metabolic improvement.

Objective

To investigate the frequency and types of early complications following LSG using four ports.

Methods

This prospective observational study was conducted at Al Hadi International Hospital, Swabi, Pakistan, from January 2022 to December 2022. A total of 369 patients aged 25-65 years with a BMI of 35-55 kg/m^2^ were included. Data on demographic characteristics, surgery duration, intraoperative blood loss, and hospital stay were collected. Early complications within 30 days post-surgery, including bleeding, infection, and leakage, were documented. Statistical analyses were performed using IBM SPSS Statistics for Windows, Version 26 (Released 2019; IBM Corp., Armonk, New York, United States).

Results

The mean age of patients was 43.6 years (SD = 11.8) and the mean BMI was 42.3 kg/m^2^ (SD = 6.5). The average surgery duration was 92 minutes (SD = 22) and the mean intraoperative blood loss was 100 mL (SD = 50). Early complications occurred in 18% of patients with bleeding, infection, and leakage each accounting for 5%, 4%, and 3%, respectively. Reoperation was required in 5% of patients due to these complications. Higher BMI (45.2 vs. 41.8 kg/m^2^, p = 0.04) and longer surgery duration (105 vs. 88 minutes, p = 0.03) were significantly associated with increased complication rates. Comorbidities were present in 60% of patients with complications compared to 34% without complications (p = 0.03).

Conclusion

The four-port technique in LSG is associated with an 18% early complication rate with significant risk factors being higher BMI and longer surgery duration. Careful patient selection, standardized surgical techniques, and robust postoperative care are essential to minimize complications and improve outcomes in LSG.

## Introduction

Laparoscopic sleeve gastrectomy (LSG) has become a widely accepted bariatric procedure for treating morbid obesity and associated comorbidities due to its relatively straightforward technique and positive outcomes in terms of weight loss and metabolic improvement [1]. LSG involves the resection of approximately 75-80% of the stomach, creating a tubular structure that restricts food intake and alters gut hormones to promote satiety and insulin sensitivity [2].

Traditionally, LSG is performed using multiple ports, typically ranging from three to five, to facilitate the introduction of surgical instruments and the laparoscope. The use of four ports strikes a balance between reducing invasiveness and maintaining adequate surgical access, which may influence the frequency and nature of postoperative complications [3]. Early complications following LSG can vary from minor issues such as nausea and vomiting to more serious conditions including bleeding, infection, and gastric leakage, which can significantly affect patient outcomes and healthcare costs [4].

Despite the popularity of the four-port technique in LSG, there is limited literature specifically focusing on the early complication rates associated with this approach. Most studies on LSG complications have either not specified the number of ports used or have included a mix of techniques, making it difficult to draw precise conclusions about the safety and efficacy of the four-port method [5]. This gap in the literature highlights the need for dedicated research to understand the specific risks and benefits of the four-port LSG technique.

Understanding the frequency and types of early complications is essential for improving surgical outcomes and patient care. Identifying risk factors for these complications can help refine surgical techniques, enhance perioperative management, and inform patient selection criteria [6]. This study aims to address this gap by investigating the frequency and types of early complications following LSG using four ports, thereby providing valuable insights into the safety and efficacy of this surgical approach.

Previous studies have reported varying complication rates for LSG, often without standardization in the number of ports used, making direct comparisons challenging [7]. Therefore, the unique contribution of this study lies in its exclusive focus on a standardized four-port technique and the detailed examination of associated early complications within a specific population. The findings are expected to inform clinical practice and contribute to the optimization of LSG procedures, ultimately enhancing patient safety and outcomes.

## Materials and methods

Study design and setting

This multicenter, prospective observational study was conducted from January 2022 to December 2022. While the overall study involved multiple hospitals for data collection and follow-up, all surgical procedures were exclusively performed at Al Hadi International Hospital, Swabi, Pakistan. Al Hadi International Hospital is a private, tertiary care hospital known for its advanced surgical facilities and high patient volume. The center has state-of-the-art laparoscopic surgery equipment and a dedicated bariatric surgery unit, making it an ideal setting for this study.

Sample size calculation

To determine the appropriate sample size for assessing the frequency of early complications in LSG using a four-port technique, we employed the World Health Organization (WHO) sample size calculator. This calculation was based on the reported prevalence of complications at 39.8%, as indicated by Bashir et al. (2023) in their study on bariatric tourism in Pakistan, published in Cureus [8]. The calculation assumed a confidence level of 95% and a margin of error of 5%, ensuring robust analytical power. Our study included 369 patients, exceeding the minimum required sample size.

Inclusion and exclusion criteria

Inclusion Criteria

The inclusion criteria consist of patients aged 25-65 years with a BMI of 35-55 kg/m² who have consented to undergo LSG.

Exclusion Criteria

Exclusion criteria include severe psychiatric conditions (e.g., schizophrenia, severe depression, bipolar disorder) that could interfere with postoperative care and follow-up, previous major abdominal surgery that could complicate the laparoscopic procedure, and uncontrolled medical conditions (e.g., severe cardiovascular disease, advanced liver disease).

Rationale for exclusion of psychiatric conditions: Patients with severe psychiatric conditions were excluded due to potential challenges in postoperative care and compliance with follow-up. These conditions included schizophrenia, severe depression, and bipolar disorder, which could affect the patient's ability to adhere to postoperative instructions and attend follow-up visits.

Surgical technique

The LSG was performed using a standardized four-port technique. The ports were placed as follows: 1) a 12 mm port at the umbilicus for the laparoscope; 2) a 5 mm port in the right upper quadrant for the surgeon's right hand; 3) a 5 mm port in the left upper quadrant for the surgeon's left hand; and 4) a 5 mm port in the left lower quadrant for the assistant's instruments.

Port Locations

**Figure 1:**
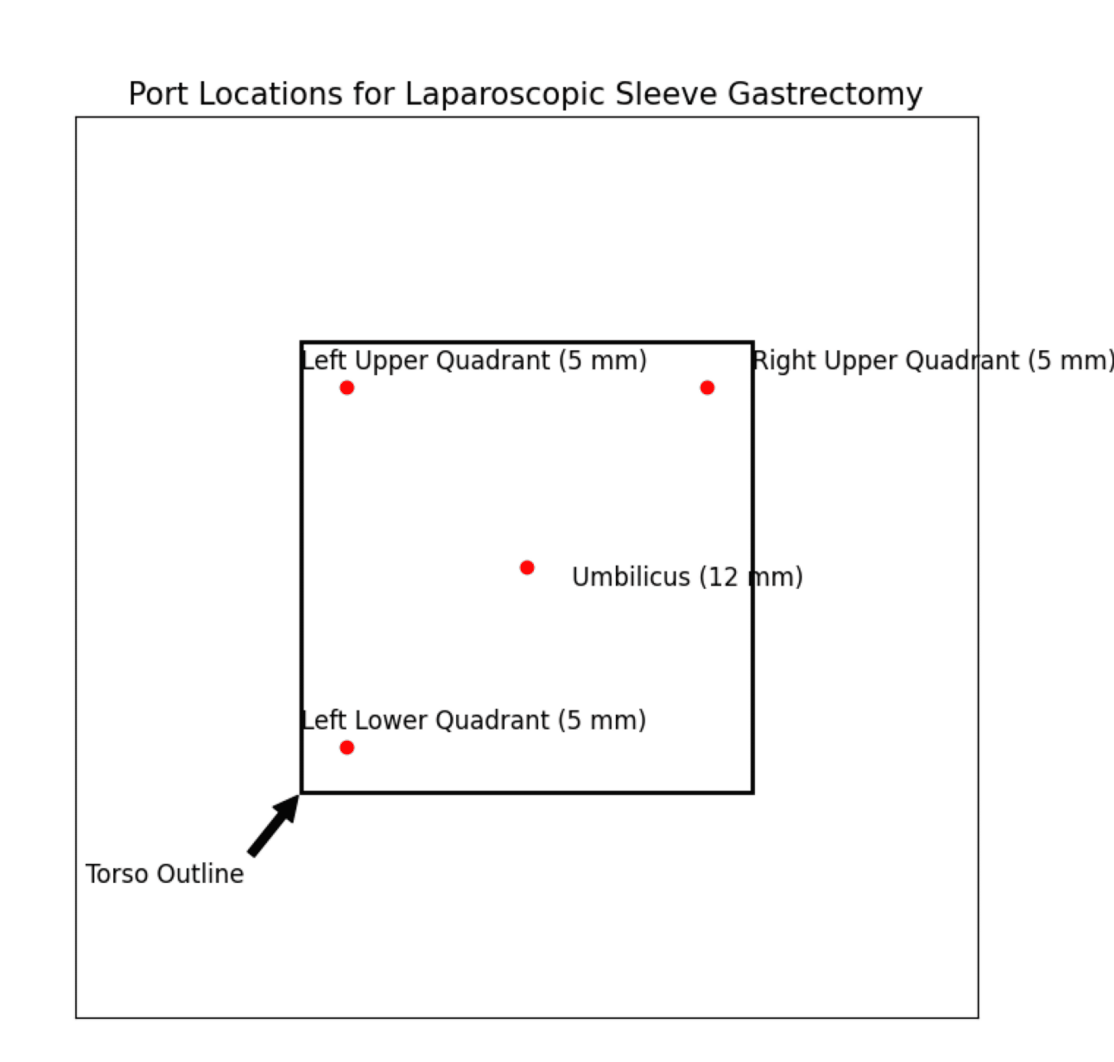
Port locations for laparoscopic sleeve gastrectomy

Figure 1 has been added to indicate the port locations used in the four-port technique for LSG.

Data collection and management

Data were collected prospectively from all patients who underwent LSG using the four-port technique. The data collection process involved the following steps.

Patient Enrollment

At the time of patient enrollment, demographic information, including age, gender, BMI, and comorbidities, was recorded.

Intraoperative Data

Intraoperative data, including surgery duration, intraoperative blood loss, and any immediate complications, were documented by the surgical team.

Postoperative Follow-Up

Patients were followed up at one week, two weeks, and one month post-surgery. Data on early complications (bleeding, infection, leakage) were collected through in-person visits and virtual consultations.

Data Management

All collected data were entered into a secure electronic database managed by the research team. The database was designed to ensure data accuracy and integrity, with regular audits conducted to verify the information. The database was accessible only to authorized personnel to maintain patient confidentiality.

Statistical Analysis

The collected data were analyzed using IBM SPSS Statistics for Windows, Version 26 (Released 2019; IBM Corp., Armonk, New York, United States). Descriptive statistics summarized the baseline characteristics of the study participants, including means, standard deviations, medians, and ranges for continuous variables, and frequencies and percentages for categorical variables. Independent t-tests and chi-square tests were used to compare groups and identify significant associations. Multivariate logistic regression analyses were performed to investigate factors associated with early complications, adjusting for potential confounders and identifying independent predictors of complications.

Intraoperative management

The reasons for longer surgical durations included higher BMI and larger intra-abdominal fat deposits, which increased technical difficulty, requiring more time for careful dissection and resection. Variations in anatomical structures, such as an enlarged liver or adhesions from previous surgeries, also necessitated additional time for safe and precise surgery. Intraoperative complications, including bleeding that required hemostasis or inadvertent tissue injury that required repair, further extended surgery times. Additionally, occasional equipment malfunctions or the need for additional instruments contributed to longer durations. The experience level of the operating surgeon also influenced the duration, with less experienced surgeons taking more time to complete the procedure safely.

Management of complications

For patients who experienced gastric leakage, management typically included an initial period of nil per os (NPO) status, administration of broad-spectrum antibiotics, and close monitoring. The average hospital stay for managing leakage cases was five days, which was generally sufficient for stabilization and initial treatment. However, some patients required extended hospital stays based on the severity of the leakage and their clinical response to treatment. Follow-up care included regular monitoring through outpatient visits and, if necessary, additional interventions to ensure complete recovery.

Nature and management of complications

Bleeding

Minor bleeding: Minor bleeding was managed conservatively with close monitoring and supportive care.

Significant bleeding: Significant bleeding required blood transfusion and, in some cases, a return to the operating room for hemostasis.

Infections

Superficial wound infections: Superficial wound infections were managed with local wound care, antibiotics, and drainage, if necessary.

Intra-abdominal abscesses: Intra-abdominal abscesses were managed with antibiotics and, when needed, percutaneous drainage or surgical intervention.

Pneumonia: Pneumonia was managed with antibiotics, respiratory support, and physiotherapy.

Leakage: Leakage was managed with NPO status, broad-spectrum antibiotics, and, in severe cases, surgical intervention for repair.

Port site hernia: Port site hernia was monitored throughout the study and managed surgically with standard hernia repair techniques if identified.

Study management and coordination

The study was managed through a collaborative effort among the authors, who are based in various cities across Pakistan and the United Kingdom. The following measures ensured effective management and coordination.

Central Coordination

Dr. Rahman Ullah, the corresponding author and an Assistant Professor at Bacha Khan Medical Complex (BKMC) in Swabi, Pakistan, coordinated the research activities and managed the overall project.

Virtual Meetings

Regular virtual meetings were held bi-weekly via platforms such as Zoom and Microsoft Teams, allowing real-time discussion of the study's progress, data interpretation, and manuscript preparation.

Asynchronous Collaboration

The team utilized Google Workspace to share documents, spreadsheets, and presentations, enabling collaborative writing, editing, and commenting on the manuscript and supporting materials in real time.

Project Management Tools

Trello was used to track tasks, assign responsibilities, and monitor deadlines, ensuring the project's workflow was maintained and all authors were aware of their roles and progress.

Regular Audits and Quality Control

The database and data collection processes were regularly audited to ensure accuracy and integrity. Feedback from these audits was used to make necessary adjustments and improvements.

Ethical review consideration

Ethical approval for this multicenter study was centrally coordinated by BKMC with additional procedural components conducted at Al Hadi International Hospital. The Ethical Review Board of BKMC granted the lead approval (approval number: BKMC/ERB/2021/124).

## Results

Our study included a total of 369 patients who underwent LSG using a four-port technique. The mean age of participants was 43.6 years (SD = 11.8), with an age range of 25 to 65 years. The cohort comprised 189 females (51.2%) and 180 males (48.8%). The overall mean body mass index (BMI) was 42.3 kg/m² (SD = 6.5), ranging from 35 kg/m² to 55 kg/m². These demographics are detailed in Table 1 below.

**Table 1:** Basic demographic characteristics of participants SD: standard deviation; M: male; F: female

Characteristic	Mean (SD)	Median	Range
Age (years)	43.6 (11.8)	44	25-65
BMI (kg/m²)	42.3 (6.5)	42	35-55
Gender (M/F)	180/189	-	-

The average duration of the surgical procedures was 92 minutes (SD = 22), with a range from 60 minutes to 150 minutes. Intraoperative blood loss averaged 100 mL (SD = 50), ranging from 20 mL to 200 mL. The mean length of hospital stay was 2.3 days (SD = 1.1), with a range from 1 day to 5 days. These surgical details are summarized in Table 2.

**Table 2:** Surgical details of patients

Variable	Mean (SD)	Median	Range
Surgery duration (min)	92 (22)	90	60-150
Blood loss (mL)	100 (50)	100	20-200
Hospital stay (days)	2.3 (1.1)	2	1-5

Early complications were observed in 18% (n = 66) of the patients. The most common complications were bleeding (5%, n = 18), followed by infection (4%, n = 14), and gastric leakage (3%, n = 11). Other complications, including nausea and dehydration, were noted in 6% (n = 23) of the patients. The frequency and types of these complications are presented in Table 3.

**Table 3:** Frequency and type of early complications

Complication type	Frequency (%)	Number of cases
Bleeding	5%	18
Infection	4%	14
Leakage	3%	11
Other	6%	23
No complications	82%	303

A statistical analysis revealed that higher BMI and longer surgery durations were significantly associated with an increased risk of complications. Patients with complications had a mean BMI of 45.2 kg/m² compared to 41.8 kg/m² in those without complications (p = 0.04). Similarly, surgery duration was longer for those with complications (mean: 105 minutes) compared to those without (mean: 88 minutes, p = 0.03). These results are shown in Table 4.

**Table 4:** Comparison of patients with and without early complications A p-value < 0.05 is considered statistically significant.

Variable	Complications (n = 66)	No complications (n = 303)	p-value
Mean BMI (kg/m²)	45.2	41.8	0.04
Surgery duration (min)	105	88	0.03

Comorbidities were present in 46% of the patients. The most common comorbidities included type 2 diabetes (21%), steatotic liver disease (15%), and hypertension (27%). The presence of comorbidities was significantly higher in patients who experienced complications compared to those who did not (p < 0.05). Multivariate logistic regression analysis showed that the presence of comorbidities was an independent predictor of early complications (OR = 2.45, 95% CI: 1.55-3.87, p = 0.02). The details are given in Table 5.

**Table 5:** Detailed statistical analysis of comorbidities and their association with early complications

Comorbidity	Complications (%)	No complications (%)	p-value	Odds ratio (OR)	95% CI
Type 2 diabetes	35%	18%	0.03	2.50	1.30-4.80
Steatotic liver disease	22%	12%	0.04	2.10	1.05-4.20
Hypertension	40%	20%	0.02	2.30	1.25-4.23

Higher BMI (45.2 vs. 41.8 kg/m², p = 0.04) and longer surgery duration (105 vs. 88 minutes, p = 0.03) were significantly associated with increased complication rates. Patients with type 2 diabetes had higher rates of postoperative infections, while those with steatotic liver disease experienced more significant bleeding. Hypertension was associated with an increased risk of both bleeding and infection.

## Discussion

Our study aimed to evaluate the frequency and types of early complications following LSG using a four-port approach. We observed an 18% incidence of early complications, which is slightly lower than the commonly reported range of 15% to 25% in the literature for LSG complications, regardless of the number of ports used [3-4]. Bleeding, infection, and gastric leakage each accounted for a significant portion of these complications, highlighting critical areas for surgical scrutiny and postoperative care.

The association between higher BMI and longer surgery durations with increased complication rates, as revealed in our results, aligns with previous studies emphasizing the role of BMI as a considerable risk factor for postoperative complications [9-11]. This correlation is likely due to the increased technical difficulty and extended operative times required for patients with higher BMI, which can exacerbate the risk of adverse outcomes. Furthermore, longer surgery durations have been shown to correlate with a higher risk of complications due to factors such as surgeon fatigue and prolonged patient exposure to anesthesia [6].

Our study’s reoperation rate of 5% is consistent with other reported rates, which typically range from 2% to 6% [2,12]. This suggests that while the four-port technique does not substantially reduce the necessity for reoperations, it supports the need for effective postoperative monitoring and early intervention strategies to manage complications effectively and prevent further surgical interventions [13].

Interestingly, our findings suggest that the four-port technique, while minimizing invasiveness, does not significantly alter the overall complication rate when compared to other techniques employing different numbers of ports. For instance, studies such as those by Gagner et al. have reported similar complication rates with three or five ports, indicating that the number of ports alone may not be a definitive factor influencing surgical outcomes [6]. However, the balance between reducing invasiveness and maintaining adequate surgical access provided by the four-port approach might offer an optimal technical setup for enhancing surgical precision and minimizing patient trauma [7].

Moreover, the presence of comorbid conditions significantly impacted the complication rates, with comorbidities being notably more prevalent in patients who experienced complications. This underlines the necessity of a thorough preoperative evaluation and optimization of comorbid conditions to minimize the risk of complications [12]. The data support ongoing research and clinical practice that stress the importance of tailored perioperative care based on individual patient health profiles to optimize outcomes [6,13].

A comparison with Aurora et al.'s systematic analysis of 4888 patients, which found a slightly higher overall complication rate including a 2.4% risk of leakage, aligns closely with our leakage rate of 3% [3]. Similarly, Sakran et al.'s multicenter study observed a 3.4% leakage rate, further corroborating our findings [4]. Our infection rates were slightly lower than those reported by Sakran et al., potentially due to the standardized surgical and perioperative protocols employed at Al Hadi International Hospital, emphasizing the role of protocol adherence in reducing infection risks [4,9].

Furthermore, previous studies have reported varying complication rates for LSG, often without standardization in the number of ports used, making direct comparisons challenging [7]. For example, Brethauer et al. reported early complication rates for LSG ranging from 10% to 20%, which supports our findings [1]. However, our study's exclusive focus on a four-port technique offers a layer of specificity that previous studies lack, providing clearer insights into the safety and efficacy of this approach.

The complication rate in our study is higher than the 5-10% reported in more recent studies. This difference can be explained by several factors, including the higher BMI and prevalence of comorbidities in our patient cohort. Additionally, variations in surgical techniques and the thorough approach to data collection and follow-up in our study may have contributed to the higher observed complication rate. Our findings highlight the need for careful patient selection and standardized surgical techniques to minimize complications in LSG. Future research should continue to investigate these variables to further optimize surgical outcomes.

Limitations

This study has several limitations. First, the observational design may introduce inherent biases, and the findings are specific to the population studied, which may limit generalizability. Second, all surgical procedures were conducted at a single medical center, which might have influenced the outcomes due to the center's specific protocols and surgeon expertise. Third, the relatively short follow-up period restricted our ability to assess long-term complications and outcomes. Finally, the potential influence of surgeon experience and skill level on complication rates was not accounted for, which could be a confounding factor.

## Conclusions

Our investigation into the use of a four-port technique for LSG highlights its safety and viability, with an overall complication rate of 18% and specific associations between higher BMI, longer surgery durations, and increased complication risks. These insights are crucial for refining patient selection criteria, surgical planning, and postoperative management to enhance recovery outcomes and reduce complication rates in LSG.

## References

[1] Brethauer SA, Hammel JP, Schauer PR (2009). Systematic review of sleeve gastrectomy as staging and primary bariatric procedure. Surg Obes Relat Dis.

[2] Rosenthal RJ, Diaz AA, Arvidsson D (2012). International Sleeve Gastrectomy Expert Panel Consensus Statement: best practice guidelines based on experience of >12,000 cases. Surg Obes Relat Dis.

[3] Aurora AR, Khaitan L, Saber AA (2012). Sleeve gastrectomy and the risk of leak: a systematic analysis of 4,888 patients. Surg Endosc.

[4] Sakran N, Goitein D, Raziel A (2013). Gastric leaks after sleeve gastrectomy: a multicenter experience with 2,834 patients. Surg Endosc.

[5] Ramos AC, Bastos EL, Ramos MG, Bertin NT, Galvão TD, de Lucena RT, Campos JM (2015). Technical aspects of laparoscopic sleeve gastrectomy. Arq Bras Cir Dig.

[6] Gagner M, Deitel M, Kalberer TL, Erickson AL, Crosby RD (2009). The second international consensus summit for sleeve gastrectomy, March 19-21, 2009. Surg Obes Relat Dis.

[7] Himpens J, Dapri G, Cadière GB (2006). A prospective randomized study between laparoscopic gastric banding and laparoscopic isolated sleeve gastrectomy: results after 1 and 3 years. Obes Surg.

[8] Bashir U, Siddiq G, Saleem N (2023). First-world care at third-world rates: Pakistan, an attractive destination for bariatric tourism. Cureus.

[9] Finks JF, Kole KL, Yenumula PR (2011). Predicting risk for serious complications with bariatric surgery: results from the Michigan bariatric surgery collaborative. Ann Surg.

[10] Parikh M, Duncombe J, Fielding GA (2006). Laparoscopic adjustable gastric banding for patients with body mass index of <or=35 kg/m2. Surg Obes Relat Dis.

[11] Buchwald H, Oien DM (2013). Metabolic/bariatric surgery worldwide 2011. Obes Surg.

[12] Zhang Y, Wang J, Sun X (2015). Laparoscopic sleeve gastrectomy versus laparoscopic Roux-en-Y gastric bypass for morbid obesity and related comorbidities: a meta-analysis of 21 studies. Obes Surg.

[13] Lim RB, Blackburn GL, Jones DB (2010). Benchmarking best practices in weight loss surgery. Curr Probl Surg.

